# Why market orientation matters for agriculture and fishery workers? Unravelling the association between households’ occupational background and caloric deprivation in India

**DOI:** 10.1186/s12889-021-10644-9

**Published:** 2021-04-08

**Authors:** Smita, Sunil Rajpal, Shiau-Yun Lu, William Joe

**Affiliations:** 1 Department of Marine Environment and Engineering, National Sun Yat-Sen Univeristy, 70 Lien Hi Road, Kaohsiung, Republic of China (Taiwan); 2 Institute of Health Management Research, IIHMR University, Jaipur, India; 3 Population Research Centre, Institute of Economic Growth, Delhi university Enclave, North Campus, Delhi, India

**Keywords:** Agriculture, Fishery, Skill, India, Calorie deprivation, Occupation

## Abstract

**Background:**

Developmental policies in low- and middle-income countries pose immense potential within the agriculture sectors to escalate economic growth and development. Almost one-half of the workforces continue to be engaged in agriculture and allied activities with a relatively lower economic contribution than those employed in other sectors. Hence, realizing such potential however requires tremendous scaling up of skill development activities in the sector. Investing in skill development of workers engaged in agricultural and allied activities can potentially display notable value additions, income generation and therefore reductions in widespread deprivations in the form of food insecurity and undernutrition. Further with the direct link between nutrition and productivity, economic gains, it is further imperative to impart market exposure among subsistence and unskilled workers. This study therefore empirically investigates the association between households’ primary occupation and caloric deprivation in India. In particular, in a multivariate and multilevel framework, we identified how closely primary occupation of households explain the variation in caloric deprivation in India.

**Methods:**

Drawing upon data from 68th round (2011–12) of nationally representative cross-sectional Household Consumer Expenditure Survey (HCES) of National Sample Survey (NSS), Government of India, we examined the association between occupational backgrounds of households and caloric deprivation (average caloric consumption as well as low calorie intake) among Indian households.

**Results:**

Evidences show that agricultural and fishery labor households have lowest calorie intake (2086 kcal) across all the occupational groups. However, market oriented skilled agricultural and fishery workers’ (2261 kcal – rural, 2165 kcal - urban) have higher calorie intakes than those belonging to subsistence agricultural (2165 kcal – rural, 2149 kcal - urban). Further, the multilevel logistic regression estimates suggest that in rural areas, households engaged in skilled agricultural and fishery works have significantly (at 5% level) lower odds ratio (OR: 0.72, with 95% CI: 0.63; 0.82) of having insufficient calorie intake compared to the unskilled agricultural and fishery laborer households. Estimates from variance partitioning based on multilevel logistic regression models suggest that the households’ occupational group accounts for 7 to 14% of total variation in calorie consumption.

**Conclusion:**

These insights when combined with the occupation-specific random-effects suggest that investing in skill development of agricultural and fishery workers may have immense potential to strengthen their nutritional status and to reduce deprivation levels.

**Supplementary Information:**

The online version contains supplementary material available at 10.1186/s12889-021-10644-9.

## Background

Given the presumptions of low-productivity, developmental policies in low- and middle-income countries usually display a pro-manufacturing bias and largely undermine the potentials within the agriculture and fishery sectors in promoting sustainable growth and development [1–5]. As a consequence, skill development is usually aligned with preferences of the manufacturing-led economic growth and is accorded high priorities in policymaking [6, 7]. For instance, in India, the thrust on such approach is evident from the creation of a separate ministry for skill development and entrepreneurship (MSDE) that is concerned with policies to impart employable skills to the working-age (15–59 years) population that accounts for about two-third of the total population. It is expected that skill development can enhance employability quotient of the labor force and thus facilitate rapid reductions in poverty and deprivation.

While about one-half of the workforce in India continue to be engaged in agriculture and allied activities [8], it contributes only one-sixth of Gross Domestic Product (GDP). This reflects an immense scope for augmenting productivity as well as value addition in the agriculture and allied sectors via promoting skill development of the workers. In the same vein, it is important to note that the success of skill development policies in India essentially hinges upon the assumption of a quick and successful structural transformation towards non-agricultural sector (manufacturing and services). But contrary to expectations, the pace of such transition has been rather slow in India. Moreover, it is unclear whether such shift away from agriculture can necessarily lead to reductions in widespread deprivations in the form of food insecurity and under nutrition. For instance, despite rapid economic growth in recent years, India is poorly ranked 97th of 118 countries in the Global Hunger Index 2016 [9]. In fact, sustained declines in nutritional intake (calories as well as other nutrients) are identified as a major developmental and food security concern [10–12].

Given the context, it is critical to unravel the association between occupational background and nutritional deprivations and thereby develop insights regarding the scope and focus of skill development policies. For instance, it is noted that poverty and nutritional deprivations are generally concentrated among households belonging to unskilled occupational categories such as agricultural labor, casual labor or fishery workers [13–18]. But it is unclear whether only selected sections or the entire agricultural and fishery sector is vulnerable to such risks. Also, there is no evidence to understand the relative advantages of skilled agricultural and fishery sector vis-à-vis other occupations. In fact, it is also feasible that market orientation of skilled agricultural and fishery sector may display favorable impact on poverty and nutritional well-being of households [19–24].

Given such possibilities, this paper aims to examine the association between occupational backgrounds with caloric deprivation (average caloric consumption as well as low calorie intake) among Indian households. As such, a focus on caloric intake is critical because it is a fundamental indicator of food security and has a major influence on economic well-being [11, 25]. Moreover, adequate calorie intake has high relevance for nutritional well-being and is regarded as a fundamental marker of public health [10, 26]. Given such relevance, an analysis of average per capita calorie intake can highlight the patterns of nutritional deprivation across various occupational groups and can effectively outline the differences and similarities therein. Thus, these results can also provide vital insights to decide upon policy approach towards skill development and diversification. In particular, this can help to comprehend whether promotion of market-oriented skills among the unskilled agricultural and fishery households can have significant influence on nutritional and food security. The relevance of these findings increases manifold because of its wider implications for other developing countries. In fact, similar patterns of income and nutritional deprivation are observed across other low- and middle-income countries and reiterate the need and relevance of a comparative analysis of nutritional well-being across various occupational groups [3, 15, 27–29].

## Methods

This study is based on data from nationally representative cross-sectional Household Consumer Expenditure Survey (HCES) conducted in 2011–12 (68th round) by National Sample Survey Office (NSSO), Ministry of Statistics and Programme Implementation (MOSPI), Government of India (NSSO 2014). The HCES is widely used by developmental practitioners and policymakers to assess the levels and patterns of food consumption across various population subgroups in India. The HCES uses a stratified, multi-stage cluster design at state-level to provide reliable estimates at state and for rural and urban areas. Households for survey are selected on the basis of circular systematic sampling. The results are estimated using data from HCES schedule 1.0 (Type 1/ Mixed Reference Period) which altogether has a sample of 1, 01,662 households (59,695 rural + 41,967 urban).

The HCES provides occupational categories of households based on the code structure provided in the National Classification of Occupations (NCO), 2004 [30]. Based on this coding structure, the households are categorized into 27 mutually exclusive occupational categories (Table 1). According to NCO 2004, market oriented skilled agricultural and fishery workers includes field crop and vegetable growers, tree and shrub growers, gardeners, horticulture and nursery growers, mixed crop growers, daily and livestock producers, poultry producers, apiarists and sericulturists, mixed animal producers, forestry workers and loggers, charcoal burners and related workers, aquatic-life cultivation workers, inland and coastal water fishery workers, deep sea fishery workers, fisherman, hunters and trappers. Further, subsistence agriculture and fishery workers also include tree trimmer, and pruner and other subsistence level agricultural and fishery workers.

**Table 1:** Description of sample population of households in India by occupational classification, National Sample Survey, 2011–2012

Household occupational groups (NCO)	Rural		Urban		All	
	N	%	N	%	N	%
Legislators & senior officials	132	0.2	217	0.6	349	0.4
Corporate managers	4144	7.3	5538	14.7	9682	10.3
General managers	40	0.1	122	0.3	162	0.2
Science professionals	137	0.2	708	1.9	845	0.9
Life science & health professionals	310	0.5	371	1.0	681	0.7
Teaching professionals	1116	2.0	1088	2.9	2204	2.3
Other professionals	1142	2.0	1536	4.1	2678	2.8
Science associate professionals	139	0.2	402	1.1	541	0.6
Life science & health associate professionals	255	0.5	351	0.9	606	0.6
Teaching associate professionals	2051	3.6	972	2.6	3023	3.2
Other associate professionals	787	1.4	1433	3.8	2220	2.4
Office clerks	1076	1.9	1746	4.6	2822	3.0
Customer services clerks	133	0.2	299	0.8	432	0.5
Personal & protective service workers	2044	3.6	2330	6.2	4374	4.6
Models, sales persons & demonstrators	3655	6.5	3906	10.4	7561	8.0
Market oriented skilled agricultural & fishery workers	15,971	28.2	1854	4.9	17,825	18.9
Subsistence agricultural & fishery workers	571	1.0	75	0.2	646	0.7
Extraction & building trades workers	4566	8.1	2608	6.9	7174	7.6
Metal, machinery & related trades workers	910	1.6	1379	3.7	2289	2.4
Precision, handicraft, printing & related trades workers	358	0.6	423	1.1	781	0.8
Other craft & related trades workers	1666	2.9	1604	4.3	3270	3.5
Stationary plant & related operators	295	0.5	332	0.9	627	0.7
Machine operators & assemblers	633	1.1	931	2.5	1564	1.7
Drivers & mobile-plant operators	2137	3.8	1950	5.2	4087	4.3
Sales & services elementary occupations	1235	2.2	1904	5.1	3139	3.3
Agricultural, fishery & related laborers	5571	9.9	820	2.2	6391	6.8
Mining, construction, manufacturing & transport laborers	5462	9.7	2722	7.2	8184	8.7
All households	56,536	100	37,621	100	94,157	100

Source: Data from National Sample Survey, Government of India

Based on a mixed recall period of 30 days and 365 days, the HCES provides direct information on household food and non-food consumption respectively. In this paper, the information on food consumption is used to estimate the average per capita per day caloric intake (in kilocalories, kcal) across households. The estimation is based on intake conversion parameter prescribed by the NSSO and which is derived from a nutrition chart that provides details regarding energy content of different foods in the Indian diet [31]. For certain food items, the intake quantity is unavailable but has been replaced by information on average energy contents per Indian rupee. It is worth noting that the consumption details are available at the household level and thus cannot be specifically associated with caloric intake of each household member. Notwithstanding this limitation, the household per capita per day calorie intake has been an important indicator to examine the levels, trends and patterns in food deprivation in India [25]. Further, we define prevalence of low-calorie intake as the situation when households are estimated to be consuming less than 80% of the prescribed calorie norms (2400 kcal in rural areas and 2100 kcal in urban areas). In other words, households with per capita per day consumption of less than 1920 kcal in rural areas and less than 1680 kcal in urban areas are regarded as undernourished household. Use of this 80% threshold is motivated by the approach adopted by NSSO in its analysis of levels and patterns in nutritional intake in India diet [31]. In fact, the estimates of caloric deprivation obtained using this benchmark is more or less similar to the proportion of nutritional deprivation estimated using other anthropometric measures such as prevalence of low body mass index among men and women in India [32]. At this point, it is worth noting that caloric deprivation should not be perceived as the measure of nutritional failure, rather it is a proxy measure to reflect the levels of nutritional deprivation. Based on this transformation of caloric information we arrive at two outcome indicators for the analysis: average per capita per day caloric intake across households (continuous outcome) and prevalence of low-caloric intake or under nutrition across households (binary outcome).

The analysis is conducted separately for rural and urban areas as they have different average calorie intake norms. It may be noted that the analysis is based on a sample of 94,157 households (56,536 – rural, 37,621 - urban) after filtering out observations where household NCO codes or other correlates are missing or not specified. Following a bivariate analysis, we use multilevel linear and logistic regression models to understand the association of occupational groups with continuous and binary outcomes, respectively. The analysis is adjusted for sampling weights as prescribed by the NSSO. For brevity, the beta coefficients and standard errors (SE) for the fully adjusted linear regression models and odds ratios (OR) and confidence interval (at 95%) (95% CI) for logistic regression model are reported. We also estimate the variance partition coefficient for both set of regressions to highlight the between-occupational group differences in calorie deprivations [33, 34]. Using the estimated variance of random effects, the variance partition coefficients (VPCs) at each level for the respective models (variation in calorie intake or variation in the Log odds of receiving insufficient caloric intake) is computed. The VPC for the concerned level is calculated by dividing the estimated variance at the concerned level by the total variance. While calculating the total variance in the logistic regression, a latent variable methods approach is used whereby the between-household variance is assumed to follow a standard logistic distribution with a value of 3.29 [33, 34]. The regression analysis is adjusted for the following indicators of households' socioeconomic status: age and sex of household head, household size, education of household head, religion, social group, wealth quintile, sampling weights and standard errors clustered at the district and state level. As a sensitivity analysis, we also ran a series of regression models taking calorie intake per consumer unit per household as outcome to account for the age and sex composition of the household members (as prescribed by NSS 2011–12) further adjusting for total land possessed by household and ration card for availing Public Distribution Services (PDS). The wealth quintiles are constructed using principle component analysis (PCA) on the 20 household durable items from NSS 68 Schedule 1.0. Such demographic and socioeconomic correlates can have significant influence on the income food and nutritional security of the households [13]. For instance, households with higher number of dependents are more likely to have lower caloric intake than those with all working members. Similarly, family with an educated household head is more likely to have adequate calorie intake. The analysis is performed in Stata 15.0 using *runmlwin* module [35–37].

## Results

**Fig. 1:**
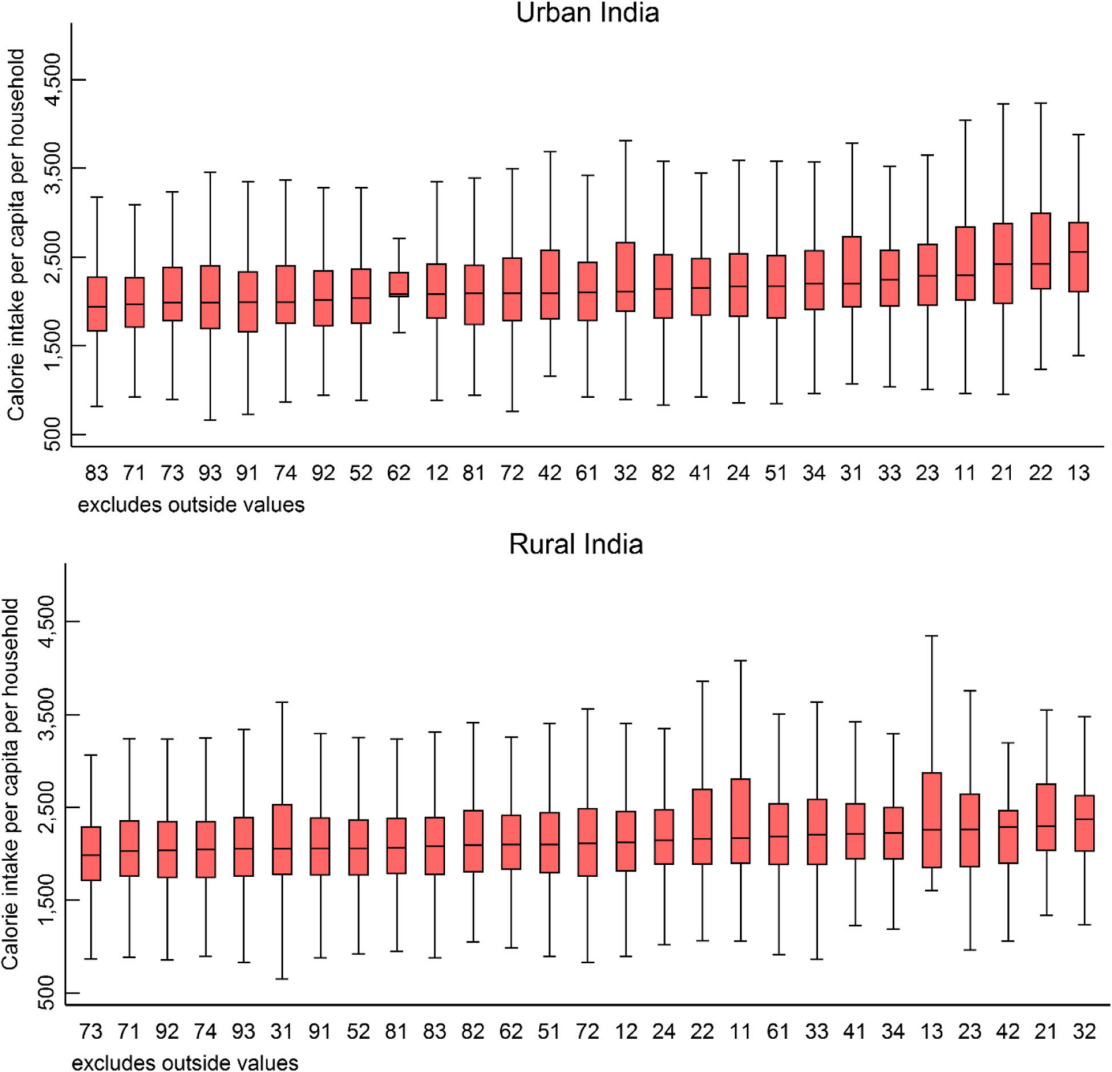
Box-plots, calorie intake per capita per household by NCO occupational classification in rural and urban India, National Sample Survey, 2011–2012. * NCO 2-digit codes: 11 Legislators and Senior Officials, 12 Corporate Managers, 13 General Managers, 21 Science Professionals, 22 Life Science and Health Professionals, 23 Teaching Professionals, 24 Other Professionals, 31 Science Associate Professionals, 32 Life Science and Health Associate Professionals, 33 Teaching Associate Professionals, 34 Other Associate Professionals, 41 Office Clerks, 42 Customer Services Clerks, 51 Personal and Protective Service Workers, 52 Models, Sales Persons and Demonstrators, 61 Market Oriented Skilled Agricultural and Fishery Workers, 62 Subsistence Agricultural and Fishery Workers, 71 Extraction and Building Trades Workers, 72 Metal, Machinery and Related Trades Workers, 73 Precision, Handicraft, Printing and Related Trades Workers, 74 Other Craft and Related Trades Workers, 81 Stationary. Plant and Related Operators, 82 Machine Operators and Assemblers, 83 Drivers and Mobile-Plant Operators, 91 Sales and Services Elementary Occupations, 92 Agricultural, Fishery and Related Labourers, 93 Mining, Construction, Manufacturing and Transport Labourers. Note: Outliers omitted for convenience of exposition

The statistical distribution of sample households across occupational groups is presented in Table 1 and across socioeconomic correlates is presented in supplementary Table S1. The average per capita calorie intake is very similar across rural (2172 kcal) and urban (2163 kcal) India (Table 2). In rural areas, households under high level services and managerial professions (particularly science, life science and health professionals) report the highest average per capita calorie intake (in excess of 2300 kcal). The lowest calorie intake (2086 kcal) is estimated for agricultural and fishery labor households. In urban areas, the similar group of professionals and managers has highest levels of caloric intake (in excess of 2400 kcal) whereas households belonging to low-end workers and laborers report low intake (below 2100 kcal). It is worth noting that across both rural and urban areas, market oriented skilled agricultural and fishery workers’ (2261 kcal – rural, 2165 kcal - urban) have higher calorie intakes than those belonging to subsistence agricultural (2165 kcal – rural, 2149 kcal - urban) and fishery workers or agricultural and fishery laborer (2086 kcal – rural, 2071 kcal - urban). Nevertheless, across both rural and urban areas, the calorie intake has a wider distribution around the mean and can be confirmed by glancing through the boxplots presented in Fig. 1 or at the standard deviations reported in Table 2.

**Table 2:** Average per capita calories consumed per day per household and the number and percentage of households with insufficient calorie intake among a nationally representative sample of households, National Sample Survey, India 2011–2012

Household occupational groups (NCO)	Calorie intake per Capita per Household				% Households having insufficient caloric intake			
	Rural		Urban		Rural		Urban	
	Mean	SD	Mean	SD	%	n	%	n
Legislators & senior officials	2382	793	2490	787	26.3	33	5.7	14
Corporate managers	2176	598	2155	553	33.8	1314	16.5	897
General managers	2377	595	2594	766	26.4	10	5.4	7
Science professionals	2420	578	2489	700	18.8	24	8.8	69
Life science & health professionals	2453	1158	2605	758	27.0	63	3.3	17
Teaching professionals	2294	618	2364	612	28.6	264	8.8	92
Other professionals	2226	576	2233	593	27.9	310	17.2	251
Science associate professionals	2200	644	2338	680	34.9	45	10.8	49
Life science & health associate professionals	2321	543	2277	619	22.0	52	14.2	49
Teaching associate professionals	2267	592	2318	690	28.0	540	13.1	109
Other associate professionals	2275	629	2296	651	23.5	195	13.0	169
Office clerks	2306	708	2243	1147	23.3	263	15.3	234
Customer services clerks	2193	444	2224	572	30.4	39	12.5	38
Personal & protective service workers	2172	607	2222	613	34.9	644	16.7	403
Models, sales persons & demonstrators	2123	568	2110	630	38.2	1238	19.8	798
Market oriented skilled agricultural & fishery workers	2261	634	2165	601	27.5	4388	18.0	323
Subsistence agricultural & fishery workers	2165	540	2149	308	33.9	175	4.6	14
Extraction & building trades workers	2096	581	2020	508	39.2	1652	22.8	661
Metal, machinery & related trades workers	2168	578	2146	666	34.8	301	19.6	288
Precision, handicraft, printing & related trades workers	2025	467	2119	585	45.2	151	20.3	109
Other craft & related trades workers	2099	568	2107	575	38.1	616	19.4	326
Stationary plant & related operators	2134	586	2146	578	33.5	106	21.2	50
Machine operators & assemblers	2155	548	2196	552	34.7	204	16.7	189
Drivers & mobile-plant operators	2146	564	1991	473	35.3	775	26.0	464
Sales & services elementary occupations	2128	579	2054	559	36.3	408	26.7	505
Agricultural, fishery & related laborers	2086	520	2071	504	39.3	2215	21.3	215
Mining, construction, manufacturing & transport laborers	2116	535	2110	630	38.8	2009	23.9	707
All households	2172	590	2163	636	33.8	18,034	18.5	7047

Source: Data from National Sample Survey, Government of India

Further, Table 2 reports the percentage of households with insufficient per capita calorie intake which is 33.8% in rural India and 18.5% in urban India. This percentage varied significantly across occupational groups. In rural India, the highest level of insufficiencies is noted among workers in precision, handicraft, printing and related trades (45.2% households). It may also be noted that Agricultural and fishery laborer as well as extraction workers also display higher levels of caloric deprivation (39% households). In urban areas, caloric deprivations are highly concentrated among households engaged in elementary occupation related to sales and services (27% households) whereas legislators, professionals and managers have very low estimated prevalence of caloric deprivation.

Among rural households, multilevel linear regression estimates (Table 3) indicate that compared to agricultural and fishery laborers, households of legislators and senior officials, life science and health professionals, and market oriented skilled agricultural and fishery workers have significantly higher average per capita calorie intake. While a number of other service sector professionals depict higher household calorie intake, but the differences are not statistically significant. Among urban households, a large number of households from service sector background as well as those engaged in market oriented skilled agricultural and fishery work report significantly higher levels of calorie intake. There is no significant difference in calorie intake of low-end occupations and laborers in urban or rural areas. Further in Table 3, we also present the multilevel logistic regression estimates for the association of caloric intake with occupational background while adjusting for demographic and socioeconomic factors such as age and sex of household head, household size, education of household head, religion, social group, and household wealth quintile. The estimates suggest that in rural areas, households engaged in market oriented skilled agricultural and fishery works have significantly (at 5% level) lower odds (OR: 0.72, with 95% CI: 0.63; 0.82) of insufficient calorie intake compared to the agricultural and fishery laborer households. While households with professionals and managers also depict lower odds, but the effects are significant only at 10% level. In urban areas, a similar relative advantage is discernible for market orientation and skills among agricultural and fishery worker households (OR: 0.72, with 95% CI: 0.59; 0.86). The odds of receiving insufficient calorie intake are also much lower for the service sector professionals, particularly life science and health professionals (OR: 0.33, with 95% CI: 0.13; 0.81). However, households engaged in sales and services based elementary occupations (OR: 1.35, with 95% CI: 1.04; 1.75) are 35% more likely to have insufficient caloric intake compared to the agricultural and fishery laborer households. Interestingly, those engaged in sales and services elementary occupations in urban areas have much higher odds of having insufficient caloric intake. This is perhaps due to the factors pertaining to their urban working lifestyle and age-specific behavioral aspects.

**Table 3:** Multilevel linear and logistic regression estimates for the association of household occupational group with per capita calorie consumption per day per household and having insufficient caloric intake in rural and urban India, National Sample Survey, 2011–2012

Household occupational groups (NCO)	Calorie Consumption per Capita per Household				Households having insufficient caloric intake			
	Rural		Urban		Rural		Urban	
	Coef	SE	Coef	SE	AOR	95% CI	AOR	95% CI
Agricultural, fishery & related labourers	ref	–	ref	–	ref	–	ref	–
Legislators & senior officials	277.3***	88.4	185.0***	54.8	0.56*	[0.29; 1.09]	0.50*	[0.24; 1.06]
Corporate managers	26.8	17.9	63.7**	30.9	0.94	[0.80; 1.12]	0.91	[0.69; 1.20]
General managers	91.1	94.4	303.4***	79.7	1.36	[0.46; 4.00]	0.50	[0.20; 1.21]
Science professionals	109.3	73.3	200.4***	32.2	0.58*	[0.31; 1.07]	0.79	[0.50; 1.24]
Life science & health professionals	178.4**	86.1	299.2***	35.6	0.90	[0.53; 1.53]	0.33**	[0.13; 0.81]
Teaching professionals	16.8	38.2	110.5***	28.9	1.06	[0.81; 1.39]	0.68*	[0.45; 1.03]
Other professionals	6.7	24.1	39.5	26.8	0.91	[0.72; 1.16]	1.16	[0.88; 1.55]
Science associate professionals	7.8	72.8	87.8*	52.1	0.95	[0.57; 1.58]	0.71	[0.41; 1.22]
Life science & health associate professionals	66.9	55.9	72.4*	43.8	0.74	[0.43; 1.29]	0.92	[0.60; 1.41]
Teaching associate professionals	33.3	24.6	75.7**	31.9	0.96	[0.78; 1.17]	1.03	[0.71; 1.49]
Other associate professionals	60.3*	35.3	85.2**	33.1	0.81	[0.60; 1.08]	0.95	[0.70; 1.29]
Office clerks	54.1	36.2	97.0**	47.5	0.77*	[0.57; 1.03]	1.03	[0.74; 1.44]
Customer services clerks	−74.4	66.9	49.9	42.2	1.08	[0.48; 2.42]	0.99	[0.59; 1.67]
Personal & protective service workers	21.3	25.2	55.1	35.6	1.00	[0.80; 1.25]	0.99	[0.71; 1.38]
Models, sales persons & demonstrators	1.8	26.9	10.9	32.0	1.08	[0.91; 1.28]	1.11	[0.83; 1.49]
Market oriented skilled agricultural & fishery workers	122.2***	19.2	137.4***	29.0	0.72***	[0.63; 0.82]	0.72***	[0.59; 0.86]
Subsistence agricultural & fishery workers	3.4	31.9	29.6	35.4	1.10	[0.80; 1.52]	0.97	[0.42; 2.20]
Extraction & building trades workers	−3.8	19.7	8.8	26.3	1.02	[0.86; 1.21]	1.01	[0.78; 1.31]
Metal, machinery & related trades workers	10.4	30.3	20.7	33.1	1.03	[0.84; 1.27]	1.13	[0.80; 1.62]
Precision, handicraft, printing & related trades workers	−63.5	45.0	−23.2	59.0	1.40	[0.87; 2.26]	1.21	[0.89; 1.66]
Other craft & related trades workers	−15.4	27.0	43.1	34.2	1.08	[0.90; 1.30]	0.87	[0.63; 1.22]
Stationary plant & related operators	−0.5	40.7	31.1	40.7	0.93	[0.54; 1.60]	1.10	[0.71; 1.71]
Machine operators & assemblers	38.1	39.2	51.5**	25.5	0.94	[0.68; 1.30]	0.99	[0.74; 1.33]
Drivers & mobile-plant operators	21.9	38.4	−0.2	31.1	0.90	[0.70; 1.17]	1.12	[0.83; 1.51]
Sales & services elementary occupations	−3.5	27.0	−19.8	29.6	0.98	[0.80; 1.21]	1.35**	[1.04; 1.75]
Mining, construction, manufacturing & transport labourers	−6.2	18.9	25.1	28.2	1.11	[0.94; 1.30]	1.03	[0.83; 1.28]

Notes: The models are adjusted for age and sex of household head, household size, education of household head, religion, social group, wealth quintile, sampling weights and standard errors clustered at the district and state level

*AOR* Adjusted Odds Ratio

^*^ *p* < 0.05, ^**^ *p* < 0.01, ^***^ *p* < 0.001

To account for the variations in the age and sex composition of the household members, Table S2 present regression estimates taking calorie intake per consumer unit as outcome variable. The estimates pattern was consistent across these models as well. For example, compared to agricultural, fishery and related laborer, the average calorie intake per consumer unit per household was significantly higher among those workers with market orientation skills in both rural as well as urban areas (Table S2). Logistic models also suggest that households engaged in market oriented agricultural and fishery works have significantly lower odds of caloric deprivation per consumer unit both in rural (OR: 0.80; 95% CI: 0.68; 0.94) as well as urban (OR: 0.78; 95% CI: 0.62; 0.99) areas. Even after adjusting for households’ landholding, the estimates were consistent, However, the estimates suggest that higher landholding of household is significantly associated with lesser probability of caloric deprivation. Among socioeconomic correlates, households’ wealth status, and education of household head was observed to be positively associated with caloric intake. Households with highly educated and female head were observed to have significantly higher calorie intake.

For rural and urban India, Table 4 presents the variance partition coefficients (VPC) for the multilevel linear regression model for average per capita calorie intake and multilevel logistic regression model for households having insufficient caloric intake. The models use five levels wherein the nesting runs in a hierarchical manner starting from households, occupational groups, districts, region and state of residence. The VPC can reveal the between-group variations attributable at the various levels. In this regard, the null model for average per capita calorie consumption in rural India shows that 10.2% of the total variance in this indicator is attributable to differences in occupational groups whereas state-related differences account for 7.4% of the variation in calorie intake. After adjusting of demographic and socioeconomic factors, the VPC of occupational groups declines to 7.8%. However, in urban areas, a greater proportion of variability in calorie intake is attributable to occupational group related differences (VPC 18.1% null model: VPC 14.8% fully adjusted model). The geographic boundaries of states and districts have low relevance in explaining variability across urban areas. In fact, region of residence has very low relevance in explaining variations in either outcome across rural or urban India. Further, the VPCs from logistic regression for households having insufficient caloric intake also present similar insights.

**Table 4:** Variance estimates and variance partition coefficients [in parenthesis] for multilevel linear (*standard error*) and logit regressions (*95% CI*) at occupational group-, district-, region- and state-levels from null and adjusted models (National Sample Survey, India 2011–12)

Level	Rural India		Urban India	
	Null model	Fully adjusted model^a^	Null model	Fully adjusted model^a^
	Calorie Consumption per Capita per Household			
State	28,904 [7.4%]	36,781 [10.8%]	21,361 [4.4%]	20,100 [5.3%]
	*(10340)*	*(8762)*	*(8448)*	*(5963)*
Region	9777 [2.5%]	8056 [2.4%]	8173 [1.7%]	7577 [2.0%]
	*(2546)*	*(2060)*	*(3105)*	*(2772)*
District	20,352 [5.2%]	20,560 [6.0%]	17,541 [3.6%]	18,703 [4.9%]
	*(3665)*	*(3630)*	*(2800)*	*(3122)*
NCO Occupational group	39,954 [10.2%]	26,386 [7.8%]	88,193 [18.1%]	56,244 [14.8%]
	*(3467)*	*(2971)*	*(23976)*	*(19608)*
Household	293,923 [74.8%]	248,559 [73.0%]	352,001 [72.2%]	276,570 [72.9%]
	*(31788)*	*(28095)*	*(38399)*	*(37475)*
	Households with insufficient caloric intake			
State	0.216 [5.0%]	0.409 [9.2%]	0.064 [1.5%]	0.149 [3.4%]
	*(0.048; 0.384)*	*(0.202; 0.615)*	*(−0.015; 0.142)*	*(0.024; 0.274)*
Region	0.145 [3.4%]	0.143 [3.2%]	0.216 [4.9%]	0.214 [4.9%]
	*(0.056; 0.233)*	*(0.04; 0.245)*	*(0.081; 0.352)*	*(0.063; 0.364)*
District	0.253 [5.9%]	0.281 [6.3%]	0.329 [7.5%]	0.376 [8.5%]
	*(0.189; 0.316)*	*(0.216; 0.346)*	*(0.249; 0.409)*	*(0.269; 0.483)*
NCO Occupational group	0.380 [8.9%]	0.315 [7.1%]	0.483 [11.0%]	0.381 [8.6%]
	*(0.291; 0.469)*	*(0.233; 0.398)*	*(0.294; 0.672)*	*(0.185; 0.577)*

Note: To compute the total variance for the multilevel logistic regression model, we follow the latent variable methods approach and assume the between-household variation to have a the variance of a standard logistic distribution of 3.29 (Browne et al. 2005, Goldstein et al. 2002)

^a^The models are adjusted for age and sex of household head, household size, education of household head, religion, social group, wealth quintile, place of residence, sampling weights and standard errors clustered at the NCO occupational group, district, region and state level

**Fig. 2:**
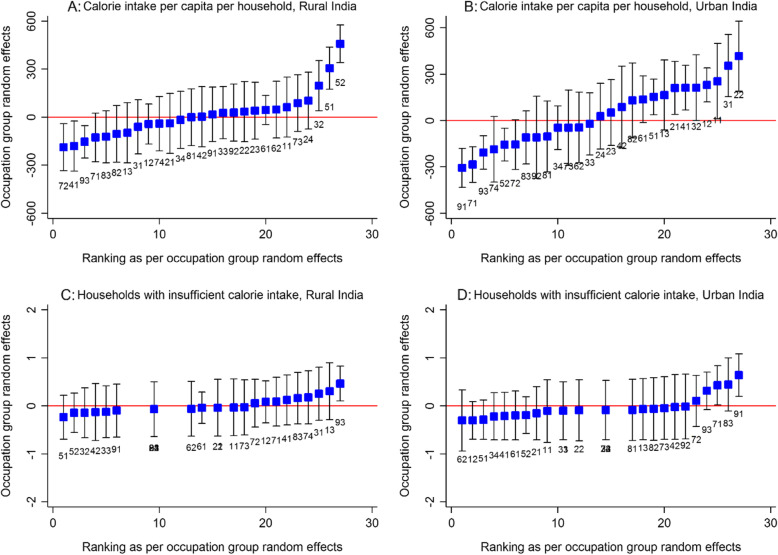
Caterpillar plot of random-intercept predictions (95% CI) versus ranking of occupation group effects for average calorie intake per capita per household and undernourished households for rural and urban India, (National Sample Survey, India 2011–12). Note: The occupation group random-effects are based on null models with sampling weights and standard errors clustered at the NCO occupational group, district, region and state level. NCO 2-digit codes: 11 Legislators and Senior Officials, 12 Corporate Managers, 13 General Managers, 21 Science Professionals, 22 Life Science and Health Professionals, 23 Teaching Professionals, 24 Other Professionals, 31 Science Associate Professionals, 32 Life Science and Health Associate Professionals, 33 Teaching Associate Professionals, 34 Other Associate Professionals, 41 Office Clerks, 42 Customer Services Clerks, 51 Personal and Protective Service Workers, 52 Models, Sales Persons and Demonstrators, 61 Market Oriented Skilled Agricultural and Fishery Workers, 62 Subsistence Agricultural and Fishery Workers, 71 Extraction and Building Trades Workers, 72 Metal, Machinery and Related Trades Workers, 73 Precision, Handicraft, Printing and Related Trades Workers, 74 Other Craft and Related Trades Workers, 81 Stationary Plant and Related Operators, 82 Machine Operators and Assemblers, 83 Drivers and Mobile-Plant Operators, 91 Sales and Services Elementary Occupations, 92 Agricultural, Fishery and Related Labourers, 93 Mining, Construction, Manufacturing and Transport Labourers

Finally, the occupational group-specific random intercepts from the four respective null models are plotted in Fig. 2. It is inferred that in rural India caloric intake of about two-thirds of the occupational groups cannot be distinguished from the overall average (Fig. 2a). However, more significant differences are apparent in urban areas (Fig. 2b). In particular, most of the legislators, professionals and managers have a higher average intake. Figure 2c and d reveal that households engaged in mining, construction, manufacturing and transport labor activities are at an elevated risk of insufficient caloric intake.

## Discussion

This analysis of nationally representative survey across India (2011–12) finds that the patterns of caloric intake and deprivations are significantly associated with occupational background of the households among both rural and urban households. Households dependent on occupations such as casual labour in agricultural and non-agricultural activities as well as those involved in low-end sales and services consumed fewer calories than others and also were at an elevated risk of caloric deprivation. In contrast, households engaged in market oriented skilled agricultural and fishery as well as the high-level professionals and managers had more than adequate calorie consumption and also were at lowest risk of such deprivations. These patterns mirror the evidence on disproportionate burden of poverty and deprivation among unskilled agricultural and non-agricultural workers in rural and urban India [13, 17, 18, 38–40].

The regressions, particularly the occupation-specific random effects (Fig. 2), reveal that market oriented skilled agricultural and fishery workers are among the select few occupations which display robust association with food and nutritional security. The households belonging to this occupational category display significantly higher levels of calorie consumption and a lower risk of caloric deprivation. The caloric intake of this group is matched only by households belonging to legislators, professionals and managers. The occupation-specific random-intercepts confirm the stark inter-occupational disparities in caloric intake with highest disadvantage for unskilled mining and construction laborers as well as those engaged in elementary sales and services workers. The between-occupation differences are also higher in urban areas. This is an important concern because higher degree of occupational diversification has not led to more equitable caloric intake although there is evidence to suggest its favorable influence on poverty reduction [41]. Also, in the urban areas, relatively lower proportion of households of same occupational group were reported to receive inadequate caloric intake. Such huge rural-urban divide in numbers perhaps display the intricacies related to market size and demand, value addition and remuneration gaps. In addition to this, factors such as accessibility and affordability to food may be at interplay for such high level of insufficiencies in rural households.

Conventionally, poverty and nutritional deprivation in India is largely discussed as a state-level phenomenon [10, 42–47]. However, there is limited evidence to understand whether it is more associated with occupational differences or other forms of disparities across states and regions. In this regard, the variance partition coefficients (VPCs) provide an overwhelming evidence to emphasize on occupations approach towards poverty and nutritional well-being. The VPCs highlight that occupational groups have the greatest effects on caloric intake across households and these effects were greater than the state-level influence. It is noted that the amount of variation in caloric intake attributable to the occupations (10.2 and 18.1% for calorie consumption in rural and urban India, respectively) is substantial even when adjusted for standard household-level socioeconomic correlates. These insights when combined with the occupation-specific random-effects suggest that policy focus to promote market oriented skilled agricultural and fishery workers can be an equally good option as direct investments in professional and managerial skills for manufacturing and services. Moreover, these findings reiterate the need for a balanced approach towards skill development in India whereby a focus on agricultural sector is not undermined because of narratives favoring manufacturing and services.

The findings are also relevant from the point of value addition and income generation capacity in agriculture and allied sector. Given the highest share in employment and a meagre contribution towards GDP of the country, the findings depict that skill development and market exposure could be an effective pathway to escalate the income generation capacity in within the sector. While occupational mobility declines with age, it is also important to impart skill and training at the right age to increase the output of skill development programmes. The findings are all more important in the light of the fact that the share of working age population in India increased to 60% of the total population in 2010 [48]. With such favorable population age-structure, a huge potential lies to boost economic growth and development via skill development [49–52]. Given the challenges pertaining to inadequate labor skills in India [53–55], recent policies have emphasized on skill development with ajor initiatives like *Pradhan Mantri Kaushal Vikas Yojana (2015)* and *Kaushal and Rozgar Mela* (2016). Our finding offers further scope for research in this domain, such as bottlenecks associated with cohorts of older adults. In the absence on an all-encompassing approaching, it is likely that poverty and deprivation can turn out to be an inter-generational affair whereby only the young generation within poor households is presented with any potential chances to improve upon household well-being. Further research on some practices and success stories (such as *Kudumbshree,* the Kerala State Poverty Eradication Mission) can be explored as they demonstrates a gendered-approach towards market oriented and skilled agrarian workers which can be an effective approach to enhance the income and nutritional status of households [56, 57].

Besides, the findings also shed significant light on the fishery sector which relatively remains unexplored for its potential impact on nutritional and income security. It is no surprise that the policy paradigm is rather in sync with the developing world whereby poverty among small-scale fisheries has remained a neglected aspect of development [15, 38, 58–60]. Whereas there is increasing evidence to support that modernization of the fisheries sector offers tremendous potential for development and growth [61–64].

It is worth noting the limitations of the analysis that can be largely associated with the nature of survey and the data. First, given the cross-sectional design, the results do not necessarily reveal the casual direction of association between occupation and caloric intake even though this does not impact the results regarding occupation-specific disparities and advantages in caloric intake. Second, although the NCO 2004 classification is sufficiently disaggregated to arrive at some meaningful inferences, but further disaggregation is advisable to understand the intricacies associated with skilled occupations within agriculture and fishery sectors. In fact, in the survey the NCO 2004 codes are missing for about 7.3% households and this can have a certain influence on the relative significance of the estimates. Third, the outcome indicator of household calorie consumption does not provide adequate insights regarding individual-level differences. Besides, to some extent, this indicator marginally underestimates the total calorie intake because of non-inclusion of food consumed outside the home [65]. Finally, the regression analysis did not account for seasonal variations in availability and prices of food items which may consequently affect the calorie consumption. However, keeping the mind the research objectives, this limitation may not affect much the inferences made in the study.

## Conclusion

To summarize, the study finds that household level calorie intake for agricultural and fishery workers with market exposure and skills is significantly higher than unskilled and subsistence ones. Further, econometric estimates suggest that households with market oriented and skilled agricultural workers are less likely to succumb to caloric deprivation compared to unskilled agricultural workers’ households. These findings clearly imply a greater scope for enhancing productivity among subsistence level workers engaged in agricultural and allied activities. More importantly, nutritional adequacy further leads to higher productivity and value addition, which can create a potential cycle of high-level income in agricultural and allied sectors.

## Supplementary Information

**Additional file 1: Table S1.** Distribution of Sample Households by Socioeconomic Characteristics, India, NSS , 2011-2012. **Table S2**. Multilevel linear and logistic regression estimates for the association of household occupational group with per consumer unit calorie consumption per day per household and having insufficient caloric intake in rural and urban India, National Sample Survey, 2011-2012.

## Data Availability

The data is available publicly without any legal or ethical restrictions at official website of MOSPI: http://www.mospi.gov.in/

## Abbreviations

HCES: Household Consumer Expenditure Survey
NSS: National Sample Survey
MSDE: Ministry of skill Development and Entrepreneurship
MOSPI: Ministry of skill Development and Programme Implementation
SE: Standard Error
VPC: Variance Partition Coefficients
PCA: Principle Component Analysis
OR: Odds Ratio
CI: Confidence Interval

## References

[1] Lewis A (1955). The theory of economic growth.

[2] Dercon S, Gollin D (2014). Agriculture in African development: theories and strategies. Annu Rev Resour Econ.

[3] Bryceson DF (1996). Deagrarianization and rural employment in sub-Saharan Africa: a sectoral perspective. World Dev.

[4] Cervantes-Godoy D, Dewbre J. Economic Importance of Agriculture for Poverty Reduction: OECD Food, Agriculture and Fisheries Working Papers, No. 23, OECD Publishing; 2010.

[5] Byerlee D, De Janvry A, Sadoulet E (2009). Agriculture for development: toward a new paradigm. Annu Rev Resour Econ..

[6] Redding S, Schott PK (2003). Distance, skill deepening and development: will peripheral countries ever get rich?. J Dev Econ.

[7] Kremer M (1993). The O-ring theory of economic development. Q J Econ.

[8] ILO (2016). India Labour Market Update, ILO country office for India, International Labor Organization.

[9] von Grebmer K, Bernstein J, Nabarro D, Prasai N, Amin S, Yohannes Y, et al. 2016 Global hunger index: getting to zero hunger. Bonn and Dublin: Welthungerhilfe, international food policy research institute, and concern worldwide; 2016.

[10] Deaton A, Drèze J. Food and nutrition in India: facts and interpretations. Econ Polit Wkly. 2009;44(7):42–65.

[11] Patnaik U. The republic of hunger. Soc Sci. 2004;32(9):9–35.

[12] Patnaik U. Neoliberalism and rural poverty in India. Econ Polit Wkly. 2007;42(30);3132–50.

[13] Sundaram K, Tendulkar SD (1990). Poverty among social and economic groups in India in. Econ Polit Wkly.

[14] Nayak P, Oliveira L, Berkes F. Resource degradation, marginalization, and poverty in small-scale fisheries: threats to social-ecological resilience in India and Brazil. Ecol Society. 2014;19(2):73.

[15] Béné C (2003). When fishery rhymes with poverty: a first step beyond the old paradigm on poverty in small-scale fisheries. World Dev.

[16] Gillespie S, Harris J, Kadiyala S (2012). The agriculture-nutrition disconnect in India: what do we know? Technical report.

[17] Parthasarathy G (1996). Recent trends in wages and employment of agricultural labour. Indian J Agric Econ.

[18] Rajuladevi AK. Food poverty and consumption among landless labour households. Econ Polit Wkly. 2001;36(28):2656–64.

[19] Irz X, Lin L, Thirtle C, Wiggins S (2001). Agricultural productivity growth and poverty alleviation. Development Policy Rev.

[20] Cecchini S, Scott C (2003). Can information and communications technology applications contribute to poverty reduction? Lessons from rural India. Inform Technol Dev.

[21] Dorward A, Kydd J, Morrison J, Urey I (2004). A policy agenda for pro-poor agricultural growth. World Dev.

[22] Ruel MT, Alderman H, Maternal and Child Nutrition Study Group (2013). Nutrition-sensitive interventions and programmes: how can they help to accelerate progress in improving maternal and child nutrition?. Lancet.

[23] Prein M, Ahmed M (2000). Integration of aquaculture into smallholder farming systems for improved food security and household nutrition. Food Nutr Bull.

[24] Bryce J, Coitinho D, Darnton-Hill I, Pelletier D, Pinstrup-Andersen P, Maternal and Child Undernutrition Study Group (2008). Maternal and child undernutrition: effective action at national level. The Lancet.

[25] Dreze J, Sen A (1989). Hunger and public action.

[26] Paul VK, Sachdev HS, Mavalankar D, Ramachandran P, Sankar MJ, Bhandari N, et al. Reproductive health, and child health and nutrition in India: meeting the challenge. Lancet. 2011;377(9762):332–49. 10.1016/S0140-6736(10)61492-4. 10.1016/S0140-6736(10)61492-4 PMC3341742 21227494

[27] Diao X, Hazell P, Thurlow J (2010). The role of agriculture in African development. World Dev.

[28] Garrity DP, Akinnifesi FK, Ajayi OC, Weldesemayat SG, Mowo JG, Kalinganire A, et al. Evergreen agriculture: a robust approach to sustainable food security in Africa. Food Security. 2010;2(3):197–214. 10.1007/s12571-010-0070-7.

[29] Zezza A, Tasciotti L (2010). Urban agriculture, poverty, and food security: empirical evidence from a sample of developing countries. Food Policy.

[30] NCO (2004). National Classification of Occupations 2004, Directorate general of employment, Ministry of Labour & employment, Government of India.

[31] NSSO (2014). Nutritional intake in India 2011–12, NSS 68^th^ round, National Sample Survey Office, Ministry of Statistics and Programme Implementation, Government of India.

[32] IIPS & Macro International (2007). India National Family Health Survey (NFHS-3), 2005–06 (Vol. 1).

[33] Browne WJ, Subramanian SV, Jones K, Goldstein H (2005). Variance partitioning in multilevel logistic models that exhibit over dispersion. J Royal Stat Society.

[34] Goldstein H, Browne W, Rasbash J (2002). Partitioning variation in multilevel models. Understanding Statistics: Statistical Issues in Psychology, Education, and the Social Sciences.

[35] Stata C. Stata Statistical Software: Release 9, StataCorp LP. TX: College Station; 2005.

[36] Leckie G, Charlton C (2013). Runmlwin-a program to run the MLwiN multilevel modeling software from within stata. J Stat Softw.

[37] Charlton C, Rasbash J, Browne WJ, Healy M, Cameron B (2017). MLwiN Version 3.00. Centre for Multilevel Modeling, University of Bristol.

[38] Kurien J. The Kerala model: its central tendency and the outlier. Soc Sci. 1995:70–90.

[39] Mehta AK, Shah A (2003). Chronic poverty in India: incidence, causes and policies. World Dev.

[40] Dev SM (2010). Inclusive growth in India: agriculture, poverty and human development.

[41] Dubey A, Gangopadhyay S, Wadhwa W (2001). Occupational structure and incidence of poverty in Indian towns of different sizes. Rev Dev Econ.

[42] Datt G, Ravallion M (1998). Why have some Indian states done better than others at reducing rural poverty?. Economica.

[43] Datt G (1998). Poverty in India and Indian states: an update. Indian J Labour Econ.

[44] Ravallion M, Datt G (2002). Why has economic growth been more pro-poor in some states of India than others?. J Dev Econ.

[45] Dev SM, Ravi C. Poverty and inequality: All-India and states, 1983–2005. Econ Polit Wkly. 2007:509–21.

[46] Bhanumurthy NR, Mitra A (2004). Economic growth, poverty and inequality in Indian states in the pre-reform and reform periods. Asian Dev Rev.

[47] Meenakshi JV, Vishwanathan B. Calorie deprivation in rural India, 1983-1999/2000. Econ Polit Wkly. 2003:369–75.

[48] United Nations (2015). Department of Economic and Social Affairs, Population Division World Population Prospects: The 2015 Revision, Methodology of the United Nations Population Estimates and Projections.

[49] Bloom DE, Williamson JG (1998). Demographic Transitions and Economic Miracles in Emerging Asia. World Bank Econ Rev.

[50] Bloom DE, Canning D, Sevilla J (2003). The demographic dividend: a new perspective on the economic consequences of population change, population matters monograph MR-1274.

[51] Mason A, Lee R (2006). Reform and support systems for the elderly in developing countries: Capturing the second demographic dividend. Genus.

[52] James KS. Glorifying Malthus: Current debate on ‘demographic dividend’ in India. Econ Political Wkly. 2008:63–9.

[53] Chandrasekhar CP, Ghosh J, RoyChowdhury A (2006). The Demographic Dividend and Young India’s Economic Future. Econ Political Wkly.

[54] Bloom DE (2011). Population dynamics in India and implications for economic growth, PGDA working paper 65.

[55] Thomas JJ (2014). The demographic challenge and employment growth in India. Econ Polit Wkly.

[56] Oommen M (2008). A micro finance and poverty alleviation: the case of Kerala’s Kudumbashree.

[57] Williams G, Thampi BV, Narayana D, Nandigama S, Bhattacharyya D (2011). Performing participatory citizenship–politics and power in Kerala’s Kudumbashree programme. J Dev Stud.

[58] Béné C, Hersoug B, Allison EH (2010). Not by rent alone: analyzing the pro-poor functions of small scale fisheries in developing countries. Devel Policy Rev.

[59] Béné C, Friend RM (2011). Poverty in small-scale fisheries: old issue, new analysis. Prog Dev Stud.

[60] Joshi PK, Gulat A, Birthal PS, Tewari L. Agriculture diversification in South Asia: patterns, determinants and policy implications. Econ Polit Wkly. 2004:2457–67.

[61] Roemer M. Fishing for growth: export-led development in Peru, 1950–1967: Harvard University Press; 1970. 10.4159/harvard.9780674418417.

[62] Thorpe A, Ibarra AA, Reid C. The new economic model and marine fisheries development in Latin America. World Dev. 2000;28(9):1689–702. 10.1016/S0305-750X(00)00045-0.

[63] Ibarra AA, Reid C, Thorpe A (2000). The political economy of marine fisheries development in Peru, Chile and Mexico. J Latin Am Studies.

[64] Golub S, Varma A (2014). Fishing exports and economic development of least developed countries.

[65] Smith LC (2015). The great Indian calorie debate: explaining rising undernourishment during India’s rapid economic growth. Food Policy.

