# Supplementary material for: Why market orientation matters for agriculture and fishery workers? Unravelling the association between households’ occupational background and caloric deprivation in India
Source: BMC Public Health. 2021 Apr 8;21:681. doi: 10.1186/s12889-021-10644-9 (PMC8028111; doi:10.1186/s12889-021-10644-9)
Supplement: Supplementary file 1 — Additional file 1: Table S1. Distribution of Sample Households by Socioeconomic Characteristics, India, NSS , 2011-2012. Table S2. Multilevel linear and logistic regression estimates for the association of household occupational group with per consumer unit calorie consumption per day per household and having insufficient caloric intake in rural and urban India, National Sample Survey, 2011-2012. [file 12889_2021_10644_MOESM1_ESM.docx]

**Table S1:** Distribution of Sample Households by Socioeconomic Characteristics, India, NSS , 2011-2012.

| Background Characteristics | Rural | | Urban | |
| --- | --- | --- | --- | --- |
|  | N (Sample) | % | N (Sample) | % |
| **Social group** | | | | |
| Schedule Tribes | 10,001 | 16.76 | 3,626 | 8.64 |
| Schedule Castes | 10,192 | 17.08 | 5,502 | 13.11 |
| Other Backward Class | 23,756 | 39.8 | 16,157 | 38.51 |
| Others | 15,733 | 26.36 | 16,674 | 39.74 |
| **MPCE Quintiles** | | | | |
| Poorest | 10,260 | 17.6 | 1,862 | 4.53 |
| Poorer | 11,014 | 18.9 | 3,805 | 9.25 |
| Middle | 13,339 | 22.89 | 6,151 | 14.96 |
| Richer | 12,865 | 22.07 | 10,693 | 26.01 |
| Richest | 10,804 | 18.54 | 18,602 | 45.25 |
| **Religion** | | | | |
| Hindu | 45,600 | 76.4 | 31,458 | 74.96 |
| Muslim | 7,043 | 11.8 | 6,093 | 14.52 |
| Others | 7,045 | 11.8 | 4,413 | 10.51 |
| **Female head of HH** | | | | |
| No | 53,246 | 89.2 | 36,655 | 87.35 |
| yes | 6,445 | 10.8 | 5,309 | 12.65 |
| **Education- Head of HH** | | | | |
| Not literate | 17,247 | 28.89 | 6,497 | 15 |
| Literate Without Formal Schooling | 387 | 0.65 | 191 | 0.45 |
| Primary | 15161 | 25.4 | 7673 | 18 |
| Secondary | 17516 | 29.34 | 13163 | 31 |
| Higher | 9,380 | 15.72 | 14,440 | 34 |
| All | 59,691 | 100 | 41,964 | 100 |

**Table S2**: Multilevel linear and logistic regression estimates for the association of household occupational group with per consumer unit calorie consumption per day per household and having insufficient caloric intake in rural and urban India, National Sample Survey, 2011-2012.

| Household occupational groups (NCO) | Calorie Consumption per Consumer Unit per Household | | | | Households having insufficient caloric intake | | | |
| --- | --- | --- | --- | --- | --- | --- | --- | --- |
|  | Rural | | Urban | | Rural | | Urban | |
|  | Coef | SE | Coef | SE | AOR | 95% CI | AOR | 95% CI |
| Agricultural, fishery & related labourers | ref | - | ref | - | ref | - | ref | - |
| Legislators & senior officials | 170.8*** | 67.7 | 182.9 | 56.2 | 0.77 | [0.50;1.19] | 0.56* | [0.30;1.04] |
| Corporate managers | 33.8 | 16.6 | -8.5 | 28.6 | 0.88** | [0.80;0.97] | 0.82 | [0.67;1.00] |
| General managers | 340.6 | 121.7 | 290.2*** | 70.4 | 1.15 | [0.54;2.45] | 0.45* | [0.21;0.97] |
| Science professionals | 104.5 | 66.1 | 91.1** | 38.8 | 0.56*** | [0.35;0.90] | 0.82 | [0.59;1.14] |
| Life science & health professionals | 120.4** | 45.4 | 182.9*** | 46.3 | 0.71* | [0.52;0.95] | 0.40** | [0.23;0.67] |
| Teaching professionals | -6.6 | 26.8 | 26.6 | 35.3 | 0.92 | [0.78;1.08] | 0.68** | [0.51;0.92] |
| Other professionals | -9.2 | 26.0 | -35.6 | 33.0 | 0.86 | [0.73;1.00] | 0.99 | [0.78;1.26] |
| Science associate professionals | -142.5 | 66.4 | 30.4 | 45.0 | 1.15 | [0.78;1.72] | 0.82 | [0.56;1.19] |
| Life science & health associate professionals | -10.7 | 49.4 | -49.6 | 47.1 | 0.78 | [0.57;1.07] | 1.00 | [0.68;1.45] |
| Teaching associate professionals | -17.4 | 21.9 | 14.7 | 36.2 | 0.92 | [0.81;1.05] | 0.88 | [0.66;1.17] |
| Other associate professionals | 80.3** | 30.2 | 16.0 | 33.4 | 0.78 | [0.64;0.94] | 0.77 | [0.59;1.00] |
| Office clerks | 30.2 | 26.4 | -17.3 | 32.3 | 0.80*** | [0.68;0.94] | 0.87 | [0.68;1.10] |
| Customer services clerks | -127.7 | 67.3 | -9.6 | 49.4 | 1.14 | [0.76;1.71] | 0.93 | [0.61;1.40] |
| Personal & protective service workers | 35.6 | 20.3 | -31.7 | 30.3 | 0.92 | [0.82;1.04] | 0.93 | [0.75;1.16] |
| Models, salespersons & demonstrators | -2.0 | 17.0 | -64.8 | 29.0 | 0.98 | [0.89;1.08] | 1.02 | [0.84;1.26] |
| Market oriented skilled agricultural & fishery workers | 63.1*** | 13.8 | 57.1*** | 32.5 | 0.88*** | [0.82;0.96] | 0.78*** | [0.62;0.99] |
| Subsistence agricultural & fishery workers | -29.9 | 36.6 | -126.3 | 89.0 | 1.08 | [0.87;1.33] | 1.11 | [0.60;2.08] |
| Extraction & building trades workers | -12.2 | 15.6 | -68.2 | 29.5 | 0.91* | [0.83;0.99] | 0.97 | [0.79;1.19] |
| Metal, machinery & related trades workers | 7.4 | 27.5 | -60.3 | 32.7 | 0.93 | [0.79;1.09] | 0.99 | [0.78;1.25] |
| Precision, handicraft, printing & related trades workers | 4.5 | 41.5 | -132.0 | 43.8 | 1.08 | [0.86;1.37] | 1.19 | [0.87;1.61] |
| Other craft & related trades workers | -27.0 | 21.6 | -40.2 | 31.8 | 1.03 | [0.91;1.16] | 0.82 | [0.65;1.02] |
| Stationary plant & related operators | 22.8 | 45.3 | -8.9 | 47.6 | 0.93 | [0.71;1.21] | 0.76 | [0.52;1.10] |
| Machine operators & assemblers | 16.7 | 32.3 | -42.8 | 35.5 | 0.81** | [0.67;0.98] | 0.90 | [0.70;1.16] |
| Drivers & mobile-plant operators | 1.7 | 19.8 | -56.5 | 31.0 | 0.91 | [0.81;1.02] | 0.93 | [0.75;1.16] |
| Sales & services elementary occupations | 61.4 | 24.1 | -61.4 | 30.8 | 0.89 | [0.77;1.03] | 1.15** | [0.93;1.43] |
| Mining, construction, manufacturing & transport laborers | 1.0 | 15.3 | -55.5 | 29.4 | 1.00 | [0.92;1.09] | 0.90 | [0.74;1.11] |

^*^ *p* < 0.05, ^**^ *p* < 0.01, ^***^ *p* < 0.001

Notes: The models are adjusted for age and sex of household head, household size, education of household head, religion, social group, wealth quintile, land holding, ration cards, sampling weights and standard errors clustered at the district and state level. AOR – Adjusted Odds Ratio
